# Appendicitis: What does really make the difference between private and public hospitals?

**DOI:** 10.1186/1471-227X-13-15

**Published:** 2013-07-26

**Authors:** Milton Steinman, Patrícia S Rogeri, Lia L Lenci, Clara C Kirschner, José Carlos Teixeira, Paulo David S Gonçalves, Nelson Akamine, Silvio Possa

**Affiliations:** 1 Emergency Department, Unidade de Primeiro Atendimento, Hospital Israelita Albert Einstein, Avenida Albert Einstein, 627 - 1o Andar, 05651-901, São Paulo, Brazil; 2 Hospital Municipal Moysés Deutsch, São Paulo, Brazil

**Keywords:** Appendectomy, Socioeconomic condition, Ultrasound, CT scan

## Abstract

**Background:**

Appendicitis is one of the most common surgical emergencies and is also a time-sensitive condition. Delays in treatment increase the risk of appendiceal perforation (AP), and thus AP rates have been used as a proxy to measure access to surgical care. It is very well known that in Brazil there are big differences between the public and private healthcare systems. Those differences can reflect in the treatment of what are considered simple cases, like appendicitis. As far as we know, it has no known links to behavioral or social risk factors, and has only one treatment option – appendectomy. The purpose of this study was to compare treatment received by Brazilian people, both by those who depend on the public and private healthcare system, and how it affects their outcome.

**Methods:**

Data was collected from the records of all patients submitted to appendectomy, in a public and in a private Sao Paulo city’s hospitals, during January to April of 2010.

**Results:**

Patients admitted by the public hospital present symptoms for a longer period of time than those treated by the private one. It took a significantly higher amount of time for the patients from the public hospital undergo surgery, and their length of stay is also significantly higher.

**Conclusions:**

Appendicitis in a public scenario is associated with increased time from onset of symptoms to operative intervention and the main reason is the delayed presentation. Clinical polices for abdominal pain should be instituted by the public healthcare system, based on population education, healthcare professionals training and establishment of strategies that can speed the diagnosis process up.

## Background

Socioeconomic inequities have been studied by different authors in different healthcare fields of interest [1-8] and their conclusions show that the less fortunate have more health issues. Most epidemiologic studies in appendicitis focused in the role of age, sex, hereditary and dietary influence on the incidence of appendicitis; few had examined the intricacy of the interplay between access to health care and clinical presentation and outcomes in patients who underwent appendectomies. Appendicitis outcome is a good candidate measure because it is one of the most common surgical emergencies and is also a time-sensitive condition. Furthermore, it has no known links to behavioral or social risk factors, and has only one treatment option – appendectomy.

Diagnosis of acute appendicitis is established primarily on patient’s history and physical examination supported by laboratory and imaging exams [1,9]. Delay in the diagnosis and treatment is by far the main cause of appendiceal perforation [7]. Emergency department consultation for evaluation of patients with acute appendicitis may be related to the socioeconomic status of the patient. In the USA, waiting time for consultation in the emergency department to evaluate patients with acute appendicitis is longer for those in a lower socioeconomic bracket [10,11].

The surgical intervention for acute appendicitis has been reported to vary by country, geographic regions, race, sex, seasons, immigrant and socioeconomic status [1,7,9]. The reasons for this variation are not fully understood.

The Brazilian health system is made up of a complex network of complementary and competitive service providers and purchasers, forming a public—private mix. Since 1989, all people have been entitled to free health care at primary, secondary, and tertiary level through a national health system, which means that theoretically there is an equal access to care.

Since it appears that appendicitis is a common, easily-treated health threatens, it was the purpose of this study to compare demographics, diagnosis work-up and outcomes in patients submitted to appendectomy in two different scenarios: public x private hospital.

## Methods

### Data collection

Data was collected from the records of patients subjected to appendectomy in Hospital Israelita Albert Einstein (HIAE), a private, high-complexity hospital located in one of the richest Sao Paulo’s neighborhoods, and Hospital Municipal Dr Moyses Deutsch (M’Boi Mirim), a public general hospital with medium-complexity, located at Jardim Angela district of São Paulo, one of the most deprived areas in the city.

### Database

We retrospectively reviewed HIAE’s database to identify adult patients with a diagnosis of appendicitis (International Classification of Diseases, Ninth Revision [ICD-9] codes 540.0, 540.1, 540.9) between January and April 2010. A similar review was performed at M’Boi Mirim. All patients submitted to appendectomy during the mentioned period were included in the study. Those submitted to other types of surgery, but who also had their appendices removed were excluded from the group. Data included demographics, interval between onset of symptoms and admission, imaging diagnostic work-up, interval from admission to surgery, AP perforation and length of stay.

### Statistical analysis

Numerical data (age, duration of symptoms, time of entry until surgery and length of stay) were described by medians and interquartile ranges (IQR) for the presence of asymmetry. Categorical data form described by absolute frequencies and percentages.

Comparisons between categorical variables were performed by chi-square test or Fisher’s exact tests.

To compare the age and the time of history we used nonparametric Mann-Whitney test. A comparison of time between admission and surgery and residence was controlled by the result of the perforation rates (AP rates), for the duration of symptoms by patients’ gender, age and achievement of Ultrasound and CT, using normal linear regression models that had multiple variables times as dependent variable and all independent control variables mentioned above and the hospital. To adjust the models were transformed logarithmically times to soften the symmetry of the data. The residual analysis of the adjusted models showed adequate to the assumptions of normality, homoscedasticity and independence of residuals.

Data were exported SPSS (SPSS Inc. Released 2008. SPSS for Windows, Version 17.0. Chicago: SPSS Inc.) statistical software for subsequent analysis. The analyzes were performed with SPSS (SPSS Inc. Released 2008. SPSS for Windows, Version 17.0. Chicago: SPSS Inc.) and considering statistically significant p values less than 0.05.

### Ethics

This study was performed with the approval of the Scientific Committee of the Hospital Israelita Albert Einstein.

## Results

A total of 225 patients (public = 96; private = 129) were identified for our study. During the analyzed period, there were 1,374 surgical procedures at Hospital Municipal Dr Moyses Deutsch (M’Boi Mirim) with 5,308 hospital discharges against 10,180 surgical procedures at Hospital Israelita Albert Einstein (HIAE) with 13,736 hospital discharges. Table 1 presents a profile of the patients admitted by M’Boi Mirim and HIAE. Patients from M’Boi Mirim are significantly younger than patients from HIAE; at M’Boi Mirim over 60% of patients are men, while less than 50% of the patients from HIAE belong to this gender.

**Table 1:** Demographic data and results

	**Hospital**		**P value**
	**MBoi**	**Einstein**	
Gender			0,055
Male	67 (60,4%)	69 (48,3%)	
Female	44 (39,6%)	74 (51,7%)	
Age	16 (11 - 28)	32 (15 - 44)	<0,001
Onset of symptoms	48 (24 - 72)	24 (12 - 48)	<0,001
US_CT			<0,001
None	65 (58,0%)	19 (13,3%)	
US	34 (30,4%)	67 (46,9%)	
TC	9 (8,0%)	20 (14,0%)	
US + CT	4 (3,6%)	37 (25,9%)	
Perforation rates (AP rates	62 (55,9%)	66 (46,5%)	0,011
Complication			0,024
No	92 (82,1%)	131 (91,6%)	
Yes	20 (17,9%)	12 (8,4%)	
Interval admission/appendectomy (hours)	12 (6 - 20)	9 (6 - 14)	<0,001*
LOS (days)	4 (3 -6)	1.8 (1.4 – 2.3)	<0,001*

*: P value adjusted by the anatomy result, duration of symptoms, gender, age and achievement of Ultrasound and CT.

Time interval between onset of clinical manifestations and hospital admission was longer for the public hospital than for the private hospital (p < 0.001).

Concerning diagnostic work-up HIAE performs more US and/or CT scans than M’Boi Mirim (p < 0001).

Finally, multivariate analyses were performed to verifiy relation between onset of symptoms, demographics, AP rates and diagnostic work–up. Patients at the public hospital had higher interval between admission and appendectomy (p < 0.001), higher AP rates at presentation (p < 0.05) and longer LOH than did patients at the private hospitals (p < 0.0001). Both hospitals have a very low and inexpressive rate of negative appendectomy (HIAE = 1, M’Boi Mirim = 1) according to the pathology reports.

## Discussion

The modern medicine is currently based on the biomedical model where the outcomes are primarily determined by the healthcare professionals’ action [12].

Despite its success, it is very well known that in Brazil there are big differences between the public and private healthcare systems. Those differences can reflect in the treatment of what are considered simple cases, like appendicitis. As far as we know, it has no known links to behavioral or social risk factors, and has only one treatment option – appendectomy.

Appendicitis is one of the most common surgical emergencies and is also a time-sensitive condition. Delays in treatment increase the risk of appendiceal perforation (AP), and thus AP rates have been used as a proxy to measure access to surgical care. Differences in ethnicity and socioeconomic status have led to marked differences in AP rates. However, when patients have equal access to care, these differences are eliminated [13,14]. Based upon these concepts, our aim was to analyze two different scenarios, public and private.

Brazil is a country of continental dimensions with widespread regional and social inequalities. To meet constitutional guarantee of access to care, the country established the Unified Health System, or Sistema Único de Saúde (SUS), which was based on the principles of universality, equity, integrality, and social participation. The SUS, which serves more than 192 million citizens, is supplemented by private insurers, which cover about 25 percent of Brazilians.

In our study, we also observed important differences between a public hospital and a private hospital concerning demographics, presentation, diagnosis and outcomes of patients with appendicitis who underwent appendectomy.

Our results show that patients admitted by M’Boi Mirim, a public hospital, are significantly younger and present symptoms for a longer period of time than those treated by HIAE.

In the government medical care, patient is usually first evaluated in an outpatient clinic by a general physician and referred to a hospital when the diagnosis of appendicitis is established or suspected, which may delay appendectomy. Private patient or one with private health insurance is generally seen by a physician of his choice, usually a specialist, who makes the clinical evaluation and performs appendectomy in a shorter period of time. Thus, this difference between public and private institutions may be caused by underlying socioeconomic and cultural disparities that might influence a delayed decision to be seen by a doctor, once there is no theoretical difference in access to health care. Once its clock starts then rupture, broader infection, bleeding and death are inevitable without surgery. Differences in average delay of key milestones in the disease course must account for the disparities. The milestones include first complaint of abdominal pain, parental recognition of urgency, initial seeking of professional care, performance of diagnostic procedures and/or referrals to other healthcare facilities, eventual correct diagnosis, and finally surgical intervention. Reductions in time between any of these milestones will reduce the chance of rupture. These findings emphasize the need to promote and disseminate information about abdominal pain in the public scenario.

It took a higher amount of time for the patients from the public hospital undergo surgery. Another striking difference was related to preoperative diagnostic work-up. The private hospital performs more ultrasound and computed tomography scans than the public hospital, but it does not reflect in the amount of negative appendectomy since both analyzed hospitals have an unexpressive rate of negative appendectomies.

On the other hand, when we exclude negative appendectomies, and check only perforated versus non-perforated appendicitis, we can see that almost one-third of all the surgeries performed by the public hospital are under perforated conditions.

Although some studies believe that appendicitis can be diagnosed without the assistance of any imaging test [15], other showed that CT scan can result in more precisely diagnosis [16,17] that is confirmed by our findings that people who underwent CT scans, which means those from the private healthcare system, have better outcome than those from the public system. In the government medical care, patient is usually first evaluated in an outpatient clinic by a general physician and referred to a hospital when the diagnosis of appendicitis is established or suspected, which may delay appendectomy. Although we could not find any study to corroborate our thesis, we observe on a daily basis that private patient or one with private health insurance is generally seen by a physician of his choice, usually a specialist, who makes the clinical evaluation and performs appendectomy in a shorter period of time. Further, time interval to perform preoperative exams, especially ultrasonography, is longer in the government medical care than in the private. Taken together, the diagnosis delay, the amount of time spent in hospital and the significantly higher number of perforated appendicitis in the public hospital when compared to the private one highlight that there are several branches that must be seen carefully to try to fix it or, at least, to minimize certain aspects that can be solved by healthcare providers.

Clinical polices for abdominal pain should be instituted not only in the public hospitals, but before that, in public health clinics, since most of the people who seeks for public healthcare assistance has to follow the government established flow, which means that the patient first goes to one of those centers and then is redirected to a hospital, if his/her situation is considered critical enough. It is normal to find in several clinics groups devoted to enlighten the population on different aspects of chronic and acute conditions that can impair their life quality. Following the already existing model, one of the strategies could pass by creating groups to teach the population on how important symptoms such as abdominal pain, fever, nausea and vomits are. The establishment of a practical, reliable score to classify the severity of the symptoms and the probability of appendicitis, similar to the Alvarado score [18], could also be a way to speed things up, avoiding higher length of stay and complications. Having a surgeon at the clinic or easily on-call could also be used to minimize the discomfort and postpone the diagnosis. Unfortunately, in this high-tech era, most of those clinics do not count on with imaging diagnosis equipments in their facilities, which may delay the appendicitis diagnosis, reinforcing the need of a well-establish protocol, well-trained people and a consistent score system to classify the severity of the case in clinical symptoms and regardless possible imaging diagnosis. In summary, our results confirm that socioeconomic differences influence people’s health status [2,4,5], even for what is considered a simple situation, such as appendicitis.

## Conclusion

In summary, we found a higher AP rate at the public hospital than at private hospitals, indicating delayed access to surgical care. Furthermore, private hospitals use more diagnostic work up policies to evaluate abdominal pain. We also found a longer LOH and disparities in the outcomes for patients treated at public hospital.

## Competing interests

The authors declare that they have no competing interests.

## Authors’ contributions

MS conceived the research idea and the manuscript. PSR analyzed and interpreted the data, and conceived the manuscript. LL and CCK were responsible for the data acquisition. JCT and SP conceded their hospital’s data bank for the study. PDSG and NA revised the manuscript critically and contributed with important intellectual content. All authors approved the final manuscript.

## Pre-publication history

The pre-publication history for this paper can be accessed here:

http://www.biomedcentral.com/1471-227X/13/15/prepub
